# Efficacy of serum procalcitonin to predict spontaneous preterm birth in women with threatened preterm labour: a prospective observational study

**DOI:** 10.1186/s12884-018-1696-2

**Published:** 2018-03-07

**Authors:** Guillaume Ducarme, François Desroys du Roure, Aurélie Le Thuaut, Joséphine Grange, Mathilde Vital, Jérôme Dimet

**Affiliations:** 1 Department of Obstetrics and Gynaecology, Centre Hospitalier Departemental, La Roche sur Yon, France; 2 Department of Biology, Centre Hospitalier Departemental, La Roche sur Yon, France; 3 Clinical Research Centre, Centre Hospitalier Departemental, La Roche sur Yon, France

**Keywords:** Procalcitonin, Spontaneous preterm birth, Threatened preterm labour

## Abstract

**Background:**

A hypothesis of preterm parturition is that the pathogenesis of spontaneous preterm birth (sPTB) may be associated with an inflammatory process. Based on this theory, we have hypothesized that an inflammatory biomarker, procalcitonin (PCT), may be a good predictive marker of sPTB at the admission for threatened preterm labour (TPL). The present study was aimed to investigate the association between serum PCT and sPTB in women with TPL and to evaluate whether PCT levels may predict sPTB in women with TPL within 7 or 14 days.

**Methods:**

In a prospective observational laboratory-based study, women with singleton pregnancies, TPL between 24 and 36 weeks and intact membranes, were enrolled between January 2014 and June 2016. Participants received routine medical management of TPL (tocolysis with atosiban, antenatal corticosteroids, and biological tests at admission (C-reactive protein, white blood cell count, and PCT measured on electrochemiluminescence immunoassay)). The primary endpoint was sPTB before 37 weeks of gestation. The value of serum PCT levels to predict sPTB within 7 or 14 days were evaluated using receiver-operating curves (ROC) analysis.

**Results:**

A total of 124 women were included in our study. PCT levels did not statistically differ between women with sPTB (*n* = 30, 24.2%) and controls (*n* = 94) (median in ng/mL [interquartile range]: 0.043 [0.02–0.07] compared to 0.042 [0.02–0.13], respectively; *P* = 0.56). PCT levels did not also statistically differ between women with sPTB within 7 days (*n* = 7, 5.6%) or 14 days (*n* = 12, 9.7%) after testing and controls. Moreover, subgroup analysis revealed no difference among PCT levels at admission between 24 and 28 weeks, between 28 and 32 weeks and over 32 weeks, and controls. On the basis of the receiver-operating characteristic curve, the highest sensitivity and specificity corresponded to a PCT concentration of 0.038 ng/mL, with poor predictive values for sPTB within 7 or 14 days.

**Conclusion:**

Serum PCT was not relevant to predict sPTB within 7 or 14 days in women admitted with TPL between 24 and 36 weeks, and thus it is not a suitable biological marker to confirm the hypothesis of an inflammatory process associated with preterm parturition.

**Trial registration:**

Clinicaltrials.gov (NCT01977079), Registered 24 October 2013.

## Background

Spontaneous preterm birth (sPTB) before 37 weeks’ gestation occurs in approximately 4.5% to 18% of pregnancies worldwide [1–3], still remains the leading cause of neonatal mortality and morbidity worldwide, causes over 70% of fetal death rates and approximately 50% of neonatal neural deficits [4], and has effects on survivors that may be lifelong [5]. An episode of threatened preterm labour (TPL) is a common cause of hospitalization during pregnancy with a frequency ranging from 9% to 24% [6–9]. Nevertheless, approximately 50% of these women with TPL will present sPTB [9]. The scale of the problem of prematurity, and the severity of its consequences, have spurred extensive research into potential causes and efficient predictors of sPTB [9, 10]. A hypothesis of preterm parturition is that the pathogenesis of sPTB may be associated with an inflammatory process [11–14]. Romero et al. have described the preterm parturition syndrome, a heterogeneous condition with premature labour as the common endpoint. They proposed that sPTB may be a result of pathological activation of signals that subsequently initiate labour rather than it is a process that is abnormal only in its timing [14].

Procalcitonin (PCT) is a 116-amino acid peptide, which is a good predictive marker of an inflammatory process with rapidly increased serum levels in inflammation or sepsis [15, 16]. Although serum PCT levels have been studied in pregnant women with preterm premature rupture of membranes (PPROM) [17, 18], only one publication with a small sample size (*n* = 53) has specifically evaluated PCT in women with preterm labour compared to healthy pregnant patients [19]. Based on these theories [11–14], we have hypothesized that an inflammatory biomarker may be a good predictive marker of sPTB at the admission for TPL. Thus, we aimed to evaluate the association between serum PCT levels and sPTB in women hospitalized with TPL between 24 and 36 weeks. The secondary objective was to evaluate whether serum PCT levels may predict sPTB within 7 or 14 days after testing.

## Methods

### Study design, site, aims and sample size

A prospective observational laboratory-based study took place from January 2014 to June 2016 at a tertiary care hospital with more than 2600 annual deliveries. Only one study had evaluated serum PCT concentrations in 53 women with preterm labour compared to 31 healthy pregnant women [19]. No study has specifically evaluated whether serum PCT levels may predict sPTB within 7 or 14 days in women hospitalized with TPL between 24 and 36 weeks. So, no sample size estimation has been done before the beginning of the study, and an interim analysis had been planned two years after the beginning of the study (with an estimation of 100–120 included women regarding our hospital data).

The aims were to evaluate the association between serum PCT levels and sPTB in women hospitalized with TPL between 24 and 36 weeks, and to evaluate whether serum PCT levels may predict sPTB within 7 or 14 days after testing.

### Characteristics of participants and description of methods

The study included pregnant women with a live singleton pregnancy, intact membranes, hospitalized with TPL between 24 and 36 weeks of gestation. Exclusion criteria were multiple gestations, known uterine malformation, cervical cerclage in situ, PPROM, suspected chorioamnionitis at the time of presentation, cervical dilatation of more than three centimetres, previous treatment with tocolysis within 7 days before inclusion, gestational hypertensive diseases, fetal growth restriction (defined as <10th centile for gestational age on Hadlock curves [20, 21], known congenital anomaly, white blood cell (WBC) count> 15.0 G/L [22], C-reactive protein (CRP) > 10 mg/L [23], and contra-indications for tocolysis, such as suspected intra-uterine infections or fetal distress.

TPL was defined as the presence of regular and painful uterine contractions that registered by cardiotocography (at least, 2 uterine contractions/10 min during 20 min), intact membranes before 37 weeks and ultrasound cervical length < 25 mm [24–26], and which may result in preterm birth [27, 28]. Gestational age at delivery was determined by the crown-rump length at a first-trimester ultrasound examination or by the date of last menstrual period and/or a second- or third-trimester ultrasound if the first-trimester ultrasound was not performed [29]. Premature births, by definition, occur prior to 37 completed weeks of gestation, and was further categorised into extreme prematurity (less than 28 weeks of gestation), severe prematurity (28–31 weeks), and moderate prematurity (32–36 weeks) [30].

The technique for ultrasound cervical length measurement has been described in detail in prior reports [26, 31]. Ultrasound cervical length measurements were subjected to a quality assurance protocol. Each ultrasound examination included 3 transvaginal sonographic cervical length measurements, and the shortest measurement was recorded. Fundal pressure was not used to assess the shortest cervical length. When a cervical funnel was present, the cervical length below the funnel was recorded.

For all patients recruited in to this study, a full examination was conducted by the attending physician. We performed standardized medical management of TPL between 24 and 36 weeks of gestation with intact membranes (sterile speculum examination, digital examination, sonographic cervical length measurement, tocolysis with atosiban, and intramuscular betamethasone 2 × 12 mg/24 h), according to recommendations and international clinical standards [24]. Although all women with TPL should not be treated with antibiotics, antibiotics in women with TPL were used in cases of group B streptococcal colonization. The clinicians were blinded to the PCT results, and the study did not modify patient management.

Maternal sociodemographic characteristics, clinical characteristics at admission, information regarding pregnancy follow up and standard perinatal outcomes were collected prospectively by the midwife or obstetrician and paediatrician responsible for the delivery and the child. Other data were collected by a research assistant, independent of the local medical team, from a prospectively maintained database of women who were included in the study. In addition, we routinely measured newborns’ umbilical arterial blood gases at birth. A paediatrician examined the newborn in all cases after delivery. Infants in need of close monitoring were transferred to the neonatal intensive care unit (NICU).

Blood samples on admission were collected in the polypropylene tubes were immediately centrifuged and the supernatant was then stored at − 35 °C until analysis. All analysis were done in the Department of Biology by the same biologist (FDR**)** and all PCT levels were measured in serum within 48 h after blood sampling via the immunoluminometric automatic analyser Cobas 6000 e601 (Roche Diagnostics International Ltd., Rotkreuz, Switzerland). The lower limit of detection of the assay was 0.02 ng/mL and the functional assay sensitivity was 0.06 ng/mL (Cobas 6000 e601; PCT package insert; Roche Diagnostics International Ltd). The assay sensitivity and inter- and intra-assay coefficients of variation (CVs) of the PCT kits, as reported by the manufacturers, are 4–10%. WBC count was determined automatically with XE-2100 Automated Haematology System (Sysmex Corporation, Kobe, Japan). CRP was measured quantitatively by immunoturbidometry with the Cobas 6000 c501 system (Roche Diagnostics International Ltd., Rotkreuz, Switzerland). The lowest detection limit of CRP with this method is 0.3 mg/L. In the study group, the cervicovaginal secretion was cultured for aerobic and anaerobic bacteria.

The endpoint was sPTB before 37 weeks of gestation. Women in the case group were defined as included women who were admitted with TPL between 24 and 36 weeks of gestation but presented ultimately sPTB before 37 weeks of gestation. Women in the control group were defined as included women who delivered at term (≥37 weeks of gestation).

### Statistical analysis

Demographic data were evaluated using descriptive statistics. Continuous data were described by their means ± standard deviations and compared by t-tests (or Mann-Whitney tests when appropriate); categorical data were described by percentages and compared by chi-square tests (or Fisher exact tests when appropriate). Serum PCT levels were not normally distributed and therefore are reported as medians and interquartile intervals. Univariate analysis were used to compare serum PCT level at admission for TPL and gestational age at birth, and to compare PCT level at admission and admission-to-delivery interval. Receiver operating characteristic (ROC) curves were generated to identify which cut-off of PCT value produced the best sensitivity to predict sPTB within 7 or 14 days after testing. STROBE guidelines/methodology for a prospective observational study were adhered to. Statistical analyses were performed with Statistical Package for Social Sciences (SPSS) software (version 17.0, SPSS Inc., Chicago, IL, USA). A *p*-value of < 0.05 was considered statistically significant.

## Results

**Fig. 1:**
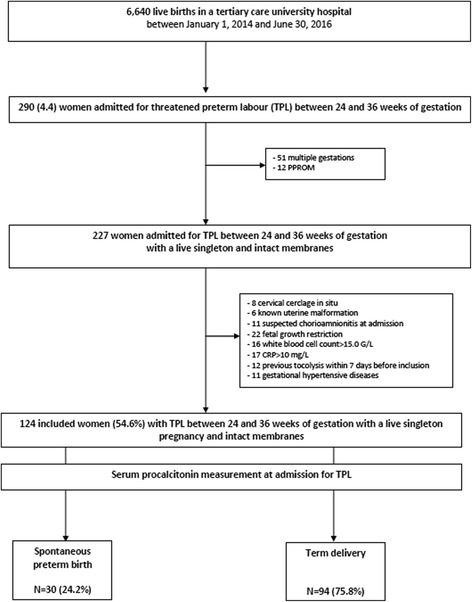
Flowchart

The study enrolled 124 patients during the study period (Fig. 1). The scheduled interim analysis showed that the magnitude of the between-group difference of serum PCT level was very small, and a sample size estimate of 7066 patients admitted with TPL should be included to show a significant difference on serum PCT levels with clinical relevance. So, the study was stopped because of negative results. Maternal and neonatal characteristics are presented in Table 1.

**Table 1:** Maternal characteristics and maternal and neonatal outcomes according to gestational age at birth

	Term delivery *n* = 94 (75.8%)	Preterm delivery n = 30 (24.2%)	*p*-value
Maternal characteristics			
Maternal age (years)	26.9 ± 4.7	28.5 ± 5.0	0.12
BMI before pregnancy (kg/m^2^)	21.9 ± 4.4	21.3 ± 3.2	0.68
Nulliparity	51 (54.3)	16 (53.3)	0.93
Variables at admission for TPL			
Gestational age at sampling (weeks)	32.5 [30.1–33.1]	32.5 [31.1–33.4]	0.48
Bishop score	3.9 ± 1.9	4.7 ± 1.9	0.08
Cervical length (mm)	19.6 ± 5.6	17.9 ± 7.3	0.20
PCT^a^ (ng/mL)	0.039 [0.030–0.050]	0.042 [0.034–0.054]	0.56
White blood cell count (× 10^9^/L)	11.0 ± 2.2	11.0 ± 2.2	0.91
C-reactive protein (mg/L)	3.8 ± 2.5	3.6 ± 2.3	0.76
Positive cervicovaginal swab cultures	5 (5.4)	5 (17.2)	0.06
Maternal outcome			
Gestational age at delivery* (w)	39.0 ± 1.2	35.0 ± 1.7	< 0.001
Interval between admission to delivery (days)	51.6 ± 18.1	21.7 ± 17.1	< 0.001
Caesarean delivery	1 (1.1)	3 (10.0)	0.04
Neonatal outcome			
Birth weight (g)	3068.3 ± 392.4	2381.2 ± 422.7	< 0.001
5 min Apgar score (points)	9.7 ± 1.1	9.4 ± 1.9	0.39
5-min Apgar score < 7	4 (4.4)	1 (3.4)	0.99
Transfer to NICU	3 (3.2)	4 (13.3)	0.06
Neonatal death	0	0	–

*TPL* Threatened preterm labour

*Gestational age at sampling (weeks) is expressed as median [interquartile range]

^a^Serum procalcitonin (PCT) level is expressed as median [interquartile range]

Continuous data are expressed as means ± standard deviations; discrete data are expressed as n or n (%). Student t test, χ2 test, non-parametric Mann-Whitney test, and Fisher’s exact test were used as appropriate. A p-value of 0.05 was considered significant

The mean age was 27.3 ± 4.8 years, and the mean gestational age at presentation was 31.7 weeks (range: 24.8–34.6 weeks). The mean gestational age at birth was 38.0 ± 2.2 weeks, and the mean interval between admission to delivery was 44.3 ± 21.9 days. Spontaneous preterm birth occurred in 30 women (24.2%). There was no statistically difference between the two groups of women, with the exception of gestational age at birth (*p* < 0.001), the interval between admission to delivery (p < 0.001), and birth weight of newborns (p < 0.001). Specifically, no difference was observed between the two groups on WBC, CRP, Bishop score and sonographic measurement of cervical length at admission (Table 1).

Only one woman delivered within 48 h. Some 12 women (9.7%) delivered within 14 days of presentation. We also found that 7 of 124 women (5.6%) delivered before 7 days from the time of admission (delivery time interval 4.4 ± 1.9 days, gestational age at birth 33.8 ± 1.2 weeks), whereas the remaining women delivered later (delivery time interval 46.7 ± 20.2 days, gestational age at birth 38.3 ± 1.9 weeks), with a statistically significant difference between gestational age at birth (*p* < 0.001).

Serum PCT levels were measurable in all samples. As shown in Table 1, serum PCT were not significantly different in women who delivered preterm (median 0.042 ng/mL, interquartile range 0.034–0.054 ng/mL) compared to women who delivered at term (median 0.039 ng/mL, interquartile range 0.030–0.050 ng/mL). Moreover, subgroup analysis revealed no significant difference among PCT levels at admission between 24 and 28 weeks, between 28 and 32 weeks, and over 32 weeks, and controls, respectively (Table 2). PCT levels did not also statistically differ between women who delivered within 7 days or 14 days after testing and the others (Table 2).

**Table 2:** Serum procalcitonin levels

	All patients (*n* = 124)	Term delivery (n = 94)	Preterm delivery (n = 30)	*p*-value
PCT level at admission for TPL(ng/mL)	0.040 [0.031–0.050] (n = 124)	0.039 [0.030–0.050] (n = 94)	0.042 [0.034–0.054] (*n* = 30)	0.56
PCT level according to gestational age at admission (ng/mL)				
Between 24 and 28 weeks	0.028 [0.020–0.038] (*n* = 8)	0.028 [0.020–0.035] (*n* = 6)	0.031 [0.020–0.042] (*n* = 2)	0.99
Between 28 and 32 weeks	0.042 [0.032–0.049] (*n* = 42)	0.040 [0.032–0.049] (*n* = 34)	0.042 [0.034–0.053] (n = 8)	0.66
> or equal to 32 weeks	0.040 [0.032–0.050] (*n* = 73)	0.040 [0.030–0.047] (n = 53)	0.041 [0.034–0.055] (*n* = 20)	0.65
PCT level according to admission-to-delivery interval (ng/mL)				
> 7 days	0.039 [0.030–0.048] (*n* = 117)	0.039 [0.030–0.047] (n = 94)	0.042 [0.034–0.054] (*n* = 23)	0.76
> 14 days	0.039 [0.030–0.049] (*n* = 112)	0.039 [0.030–0.047] (*n* = 94)	0.039 [0.030–0.054] (*n* = 18)	0.97

*TPL* Threatened preterm labour. *PCT* Procalcitonin

PCT levels are expressed as median [interquartile range]

PCT levels were not correlated to Bishop score at admission or to other routine laboratory tests (WBC and CRP) at admission for TPL. The cut-off value was then determined by application of ROC curve analysis for PCT levels against sPTB and admission-to-delivery intervals ≤7 and ≤14 days. For all of them, the highest sensitivity and specificity corresponded to a PCT concentration of 0.038 ng/mL, and the predictive values were generally poor (Table 3).

**Table 3:** The prognostic value of serum PCT determinations in the prediction of spontaneous preterm delivery and admission-to-delivery interval

PCT ≥ 0.038 ng/mL	Delivery		
	≤ 36 weeks	Within 7 days	Within 14 days
Sensitivity (%)	63.3 [43.9–80.1]	57.1 [18.4–90.1]	66.7 [34.9–90.1]
Specificity (%)	47.3 [36.9–57.9]	44.8 [35.6–54.3]	45.9 [36.4–55.7]
Positive predictive value (%)	27.9 [17.7–40.1]	5.9 [1.6–14.4]	11.8 [5.2–21.9]
Negative predictive value (%)	80.0 [67.0–89.6]	94.5 [84.9–98.9]	92.7 [82.4–98.0]
Likelihood ratio +	1.20 [0.86–1.68]	1.04 [0.53–2.01]	1.23 [0.80–1.91]
Likelihood ratio -	0.77 [0.46–1.30]	0.96 [0.40–2.30]	0.73 [0.32–1.66]

## Discussion

Our prospective, observational, laboratory-based study found that PCT levels at admission for TPL between 24 and 36 weeks of gestation did not statistically differ between women with sPTB and term birth, and serum PCT have poor predictive values for sPTB and admission-to-delivery intervals ≤7 and ≤14 days. Moreover, subgroup analysis revealed no difference among PCT levels at admission between 24 and 28 weeks, between 28 and 32 weeks and over 32 weeks, and controls.

The principal strength of this study is the exclusion of all situations which may influence the initial evaluation of women with TPL at admission and the exclusion of all situations which may also influence perinatal outcome (gestational age at birth, admission-to-delivery interval, neonatal morbidity), as multiple gestation, known uterine malformation, cervical cerclage in situ, or PPROM. However, it is important to note that patients with any biological or clinical sign of infection and/or chorioamnionitis were excluded from the study, minimizing the interference of infectious mechanisms on the timing of birth. Preterm birth may occur in one of three circumstances: iatrogenic due to fetal or maternal requirement, sPTB with intact membranes, or PPROM [30]. Then, our strict inclusion and exclusion criteria allow us to evaluate serum PCT in a homogeneous group of women admitted for TPL with intact membranes and strength our results concerning PCT levels as a potential predictive factor of sPTB. Second, our rates of sPTB before 37 weeks (24.2%) and spontaneous birth seven days following admission for TPL (5.6%) were in accordance with other published study concerning TPL in France. Pinton et al. reported rates of delivery before 37 weeks and seven days following enrolment in a prospective study in Strasbourg, France, which were 28% and 10%, respectively [32]. Third, we used a standardized measure of serum PCT in a prospective laboratory-based study with carefully characterized obstetric patients and prospectively maintained database of included women. This allowed a complete evaluation of serum PCT for women presented TPL. Four, our results concerning serum PCT levels at admission were consistent with other findings in the literature about PCT levels during normal pregnancy [33, 34]. Kucukgoz Gulec et al. evaluated PCT in 64 preeclamptic patients compared to 33 healthy pregnant patients. They reported a median PCT level at 0.040 ng/mL (interquartile range 0.002–0.075) at 35.1 ± 2.6 weeks of gestation in normal pregnancies [34].

### Limitations of the study

Our study has some limitations. First, that the study was underpowered to show a significant difference on serum PCT levels with clinical relevance. The decision to stop the study was based on the very small magnitude of the between-group difference without clinical relevance and a sample size estimate of 7066 patients with TPL who should be included to show a significance difference on serum PCT levels. Second, we only studied PCT levels as a predictive factor of sPTB without success. Several other inflammatory mediators (interleukins 6 and 8), possibly in combination, should be evaluated in these situations [35].

It is difficult to compare our results with the literature. Numerous biological mechanisms that vary between individuals may be implicated in the genesis of sPTB [36], and are poorly understood, despite significant research efforts [3, 30]. Systemic inflammatory response syndrome has been suggested as a diagnosis when no etiologic organism can be found, and infection accounted for up to 30% of cases of preterm labour may either be clinically-evident or sub-clinical. Then, this hypothesis of inflammation-infection in sPTB has been evaluated and inflammatory cytokines can be detected in elevated concentrations in the amniotic fluid and plasma of women with preterm labour [11, 19, 35]. Sorokin et al. demonstrated that an elevated maternal serum concentration of inflammatory biomarkers (interleukin 6 and CRP) are risk factors for PTB < 32 weeks [35]. Only one study has evaluated serum PCT concentrations in patients with sPTB and intact membranes [19]. The study population consisted of 53 women with preterm labour and 31 healthy pregnant women. Serum PCT concentrations were significantly higher in patients with preterm labour compared to healthy pregnant patients (1.66 compared to 1.06 ng/mL; *p* < 0.05). Although at the onset of preterm labour serum PCT level in sPTB was higher that term birth, the difference was not significant [19]. No significant differences were also observed in PCT levels according to the admission-to-delivery interval. The authors concluded that the predictive value of serum PCT for patient admitted with TPL was unsatisfactory with poor predictive values for delivery within 36 weeks, 3 days or 7 days after admission [19].

## Conclusions

Serum PCT was not relevant to predict sPTB within 7 or 14 days in women admitted with TPL, a singleton pregnancy, and intact membranes between 24 and 36 weeks. These findings suggest that PCT is not the good biological marker to confirm the hypothesis of an inflammatory process associated with preterm parturition. Another study using several other inflammatory mediators (interleukin 6), possibly in combination, should be evaluated in these situations.

## Abbreviations

CRP: C-reactive protein
NICU: Neonatal intensive care unit
PCT: Procalcitonin
PPROM: Preterm premature rupture of membranes
ROC: Receiver-operating curve
sPTB: Spontaneous preterm birth
TPL: Threatened preterm labour
WBC: White blood cell
